# What contraception do women use after experiencing complications from abortion? an analysis of cohort records of 18,688 postabortion care clients in Tanzania

**DOI:** 10.1186/s12905-018-0687-9

**Published:** 2019-01-28

**Authors:** Colin Baynes, J. Kahwa, G. Lusiola, F. Mwanga, J. Bantambya, L. Ngosso, M. Hiza

**Affiliations:** 1 EngenderHealth, 440 Ninth Avenue, New York City, NY 10001 United States; 2 EngenderHealth, Plot #254, Mwai Kibaki Road/Kiko Avenue, PO Box 78167, Dar es Salaam, Tanzania; 3 Ministry of Health, Community Development, Gender, Elderly and Children (MOHCDGEC), PO Box 9083, Dar es Salaam, Tanzania

**Keywords:** Postabortion care, Family planning, Contraceptive uptake, Decentralization, Tanzania

## Abstract

**Background:**

The family planning component of postabortion care (PAC) is critical, as it helps women to prevent unintended pregnancies and reduce future incidence of life-threatening unsafe abortion. In Tanzania, PAC was recently decentralized from tertiary-level district hospitals to primary health care dispensaries in four regions of the country. This analysis describes interventions used to improve access to high quality PAC services during decentralization; examines results and factors that contribute to PAC clients’ voluntary uptake of contraception; and develops recommendations for improving postabortion contraceptive services.

**Methods:**

This analysis uses service delivery statistics of 18,688 PAC clients compiled from 120 facilities in Tanzania between 2005 and 2014.

**Results:**

This study suggests that efforts to integrate postabortion family planning into treatment for incomplete abortion contributed to higher postabortion contraceptive uptake (86%). Results indicate that variables associated with significant differences in contraceptive uptake were facility level, age, gestational age at the time of treatment, and uterine evacuation technology used.

**Conclusion:**

The experience of expanding PAC services in Tanzania suggests that integrating contraceptive services with treatment for abortion complications can increase family planning use.

## Background

**Fig. 1:**
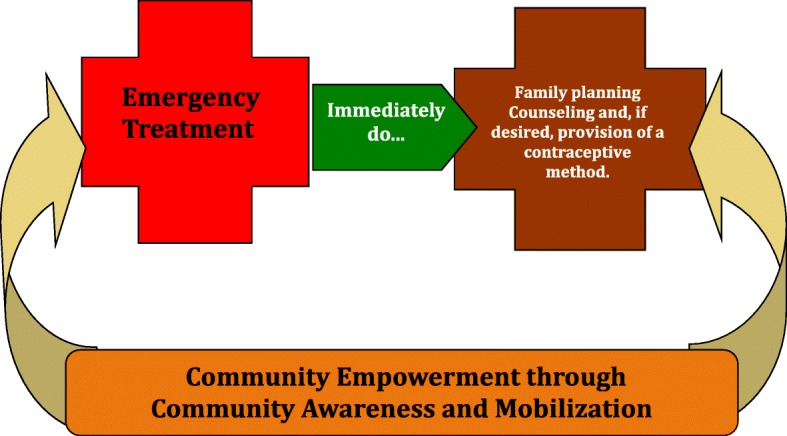
The Postabortion Care (PAC) Model

In 2012, approximately 213 million pregnancies occurred worldwide, of which 85 million (40%) were unintended. Half of these ended in induced abortions [1] . During this year, 7 million women who had induced abortions received lifesaving treatment for complications from unsafe pregnancy termination. This treatment, postabortion care (PAC), is a service package that addresses the emergency treatment and preventive health care needs of women experiencing complications from early pregnancy termination, both spontaneous and induced. This includes evacuation of residual products of conception from the uterus and family planning services (FP). The latter component of PAC includes discussion of postabortion fertility, optimal and desired timing of future childbearing after the evacuation procedure, contraception, and, if the client chooses, the actual provision of a contraceptive method. Over time, a holistic programming model was developed that includes community sensitization and mobilization to address demand-side barriers to access to PAC services (Fig. 1). In spite of global progress in this regard, estimates made by health professionals (averaged across several surveys conducted between 2000 and 2008) indicate that only 60% of women experiencing complications from abortion were expected to have accessed PAC, with the remaining two-fifths of women with postabortion complications not receiving such care [2, 3]. It follows that unsafe abortion accounts for a sizeable proportion of all maternal deaths globally (8%), mostly in developing countries [4].

Strengthening contraceptive provision during PAC is critical, as it helps women to prevent unintended pregnancies and reduce future incidence of unsafe abortion. Women seeking postabortion emergency care are particularly in need of contraceptive services: These clients are sexually active and at risk for subsequent pregnancy, not currently pregnant, and often highly desirous of avoiding childbearing. They are in contact with the health system, are often disinclined to return to the facility for a follow-up visit [5–7], and, according to global recommendations, are advised to delay a subsequent pregnancy for at least six months, for optimal health of the woman and her future newborn [8, 9].

Fertility can return 1–2 weeks after vacuum aspiration of the uterus or medical treatment for abortion complications [10, 11]. All contraceptive methods are safe to use after PAC emergency treatment, whether it is performed medically, through uterotonic drugs, via vacuum aspiration, or surgically to remove residual products of conception. When treated medically, a PAC client may start using hormonal methods—including oral contraceptives, injectables, and implants—immediately after the onset of treatment, and an intrauterine device (IUD) may be placed when it is certain that the uterus is empty. After vacuum aspiration, all methods, including IUDs and implants, may be started immediately following a first- or second-trimester procedure. Only women who have had a septic abortion or evacuation with sharp curettage should wait until they are healed before having an IUD inserted, but they may start any other method of contraception. Providing voluntary methods at the time of PAC, rather than at a follow-up visit, increases contraceptive uptake and reduces future risk of unintended pregnancy [12].

Accordingly, contraceptive services are an essential component of PAC. Various international technical working groups and organizations have concluded that a range of contraceptive methods, accurate information, counseling, and referral for ongoing care should be made available to women who have received PAC, and that this should start before they are discharged from a facility [9, 13]. The U.S. Agency for International Development (USAID) has offered guidance on high-impact practices for operationalizing these recommendations in health service delivery and management settings [14]. Furthermore, multiple studies have shown that women who receive PAC services that include FP have high contraceptive uptake [15, 16]. Studies in developed and developing countries assessing facility-level interventions, such as providing contraception in the same location and the same time as PAC, show that offering a wide range of methods and using obstetric and midwifery staff to provide contraception improves overall uptake of a contraceptive method [17].

This paper describes the process and effects of expanding access to PAC, including postabortion FP services, in mainland Tanzania. The legal status of abortion in Tanzania is ambiguous: the penal code is broadly understood to authorize abortion to save a woman’s life, but provisions for this, or allowance for it in the case of rape or incest, are not incorporated into national law that prohibits the practice. The lack of clarity creates confusion amongst providers and women who fear persecution, which, in turn, pushes women to seek abortion clandestinely. In 2013, women obtained just over 405,000 abortions, for a national rate of 36 abortions per 1000 women aged 15–49 and ratio of 21 abortions per 100 live births. For each woman treated for an induced abortion complication, 6 times as many had an abortion, but did not receive care [18]. According to the Ministry of Health, Community Development, Gender, Elderly and Children (MOHCDGEC), 19% of maternal deaths in Tanzania are due to abortion complications [19].

From 2005 to 2014, the MOHCDGEC implemented facility-based, provider-focused, and community-level health system interventions to improve the accessibility and quality of PAC services, with technical assistance from EngenderHealth, an international nongovernmental organization. Global guidance on the design and delivery of PAC was tailored into implementation approaches that were contextually appropriate to the cultural, health care delivery, and broader environmental context of rural and peri-urban Tanzania, where this work was conducted. Between 2005 and 2014, the MOHCDGEC and health care providers in Tanzania decentralized PAC from tertiary-level district hospitals to primary health care dispensaries in four regions of the country, introducing the manual vacuum aspiration (MVA) treatment approach for PAC in all settings and scaling up the availability of PAC from 11 facilities in 2005 to 239 by 2013. PAC in this situation included provision of emergency uterine evacuation services (most frequently through MVA), immediate FP counseling and, if desired, provision of a contraceptive method, and linkages through referral to other reproductive health services.

This analysis of PAC service delivery statistics, which were compiled in facility registers during this time period, has three following objectives. The first is to describe interventions used to increase access to, utilization of, and quality of PAC services during the period of decentralization. Second, it aims to examine results and factors that contribute to PAC clients’ voluntary uptake of contraception. Lastly, it develops recommendations for improving postabortion contraceptive services.

### Description of PAC program development and interventions in Tanzania

In 2005, the MOHCDGEC initiated a pilot project in Geita District (then part of Mwanza Region^1^) to demonstrate how to scale up PAC from district or referral hospitals to the level of intermediate health centers and primary-level dispensaries. The pilot comprised three elements: training service providers, setting up comprehensive PAC services and infrastructure, namely the establishment in each facility of an evacuation room where all clients admitted for routine complications from abortion (both spontaneous and induced) can receive counseling, removal of retain products in the uterus via vacuum aspiration and family planning services, including access to contraception. Of note, the evacuation methods available for clients in these settings was limited to MVA with occasional utilization of sharp curettage. Up to present, medication evacuation of the uterus, namely through misoprostol, is not permitted for PAC treatment as per MOHCDGEC policy in mainland Tanzania. Lastly, the model used to expand PAC coverage included community awareness raising and mobilization. For this, service providers, convened community meetings in their catchment communities during outreach events that centered on other primary health care issues. During these occasions, providers educated communities on complications from early termination of pregnancy and forged linkages with community health volunteers, whom educated them on FP and PAC and encouraged to convene community discussion groups on these issues. For clients that extended their permission to do so, volunteers would conduct home visits to former clients, during which they would counsel women on post-discharge self-care behaviors, FP and contraceptive methods.

Within Geita District, 11 facilities (the district hospital, seven health centers, and three dispensaries) were chosen for the pilot. Figure 1 illustrates the PAC model piloted in Geita District from 2005 to 2007.

The scale-up of the PAC model started in 2007, based on recommendations of an independent evaluation performed by the Population Council [20]. The expansion of PAC services was concentrated in Geita and three surrounding regions—Mwanza, Simiyu, and Shinyanga—and received technical, implementation, and evaluation support from EngenderHealth and funding from USAID.

The scale-up of the PAC model followed vertical and horizontal strategies. The vertical strategy, which was implemented during 2007, involved efforts to institutionalize PAC. Critical to this was the promulgation of clinical guidelines for PAC, which established MVA as the optimal clinical technique, and legislation to permit task shifting of MVA to mid-level providers, including nurses and midwives. At this time, PAC was included in the National Package of Essential Health Interventions and MVA kits in the national essential drugs and supplies list. During this period, the MOHCDGEC also, importantly, adopted of a national PAC curriculum and training guidelines. The horizontal strategy was based on district-level decentralization of PAC coverage, from district hospitals to health centers and dispensaries, in the 16 districts that comprised the four regions. With assistance from EngenderHealth, participating districts conducted assessments to identify eligible lower-level sites and planned when to devolve PAC services. Trainings in the four regions ensued for assistant medical officers, nurse midwives, and nurses to deliver MVA.

Instruction and support for facilitative supervision was an important factor in ensuring the quality of services. With this, providers in management positions at scale-up facilities learned to facilitate the quality improvement methodology known as COPE®^2^,^3^ which includes performance improvement checklists, questionnaires, and guidance on facilitating supportive supervision visits that promote development of corrective action plans in such areas as counseling, treatment procedure, infection prevention, and contraceptive services. Another important aspect of the decentralization of PAC services was the strengthening of monitoring systems. With support from EngenderHealth, the Government of Tanzania developed a PAC register that was gradually incorporated into existing health management information systems at the district—and ultimately the national—level. With this, the decentralization of PAC took place in phases in these 16 districts in Geita, Mwanza, Simiyu and Shinyanga. By 2014, PAC services eventually covered 239 sites: 15 hospitals, 67 health centers, and 157 dispensaries.

## Methods

### Data collection and management

Data were collected from 120 public-sector facilities in two of the regions where PAC was decentralized between 2005 and 2014, Geita and Mwanza. Data came from PAC registers kept in facilities’ evacuation rooms, where providers maintained records of their encounters with clients that presented to these facilities with complications from spontaneous and induced abortion. Data included service delivery details on women who received treatment for postabortion complications: demographic information; gestational age at time of abortion; the method of uterine evacuation; treatment complications; and provision of FP counseling and methods, by method type.

In order to obtain a deeper understanding of the levels of complications faced by women that reported to these facilities during this period, additional service statistics were compiled from registers in the obstetrical-gynecological wards of higher-level facilities. These sites admitted women experiencing more severe complications from abortion (spontaneous or induced) than those of women that were transferred to the evacuation rooms, which had been established during the scale up of PAC. These complications included severe bleeding, hemorrhage, sepsis, shock and uterine perforation. To identify these cases, data collectors looked at records of clients’ gestational age, considering any complication that occurred at or prior to 28 weeks gestation as abortion related. Data collectors identified 1525 cases of these complications, accordingly. The median timing of these complications was 14 weeks gestation (range was 2–24 weeks). Of these 848 were hemorrhage (56%), 433 were sepsis (28%), 146 were uterine rupture (10%) and 98 (6%) were not attributed to a particular cause. Because these data sources did not include information on contraceptive provision, in addition to the fact that above-described scale up of PAC did not emphasize treatment and FP services for women presenting with these severe morbidity, these cases were not included in the following analysis.

The register data were anonymized, so that no names or health system identification numbers of clients were collected. Other factors that may have affected FP uptake by PAC clients were unavailable from the registers used during this query. These include the availability of essential commodities, the quality of counseling, interactions with providers, clients’ knowledge and prior use of contraceptives, and clients’ fertility intentions. Register data were collected under technical assistance agreements between national authorities at the MOHCDGEC and EngenderHealth. All data were entered into the EngenderHealth/Tanzania monitoring and evaluation database and downloaded into Stata 14 for cleaning and analysis.

The Tanzanian National Institute of Medical Research and the U.S.-based Western Internal Review Board approved the study protocol.

### Data analysis

The data analysis, as presented in the section that follows, is organized according to three objectives. First, to illustrate the characteristics and profile of women admitted for PAC services in Tanzania during the period of decentralization from 2005 to 2014; second, to evaluate service delivery trends during this time frame; and, lastly, to understand the factors associated with PAC clients’ adoption of modern contraceptive methods prior to discharge from the facility.

Roughly 13.2% of all observations recorded in the dataset contained missing data on any register variable used in this analysis. For analysis related to Objective 1, we excluded missing data from the analysis, as they skewed the results of univariate methods employed to illustrate the characteristics of PAC clients. This was also done for analysis related to objectives 2 and 3, with the exception of cases when contraceptive method uptake was coded as missing. Based on programmatic experience suggesting that missing PAC FP data usually indicates that the client did not receive a method of contraception, missing data on PAC contraceptive uptake were included in these analysis, on the assumption that it meant the client did not adopt a method before discharge.

First, univariate data analysis was conducted to illustrate the social and demographic characteristics of PAC clients and key service-seeking behaviors. Second, chi-squared linear trends were analyzed to evaluate the statistical significance of trends in the utilization of PAC services. Finally, unadjusted analysis between variables of interest and outcomes of PAC FP uptake were conducted using data compiled from all years from 2005 to 2014. Bivariate associations first used chi-squared analysis; then, for all variables where associations were significant, we estimated an adjusted model using multivariate logistic regression techniques to estimate the odds of contraceptive uptake. This was done to examine for factors associated with any modern method uptake by PAC clients and then studying uptake of long-acting reversible contraceptives (LARCs) or permanent methods (PMs). The adjusted odds of method uptake did not reveal any noteworthy differences in significance or magnitude from the crude odds ratios calculated from the bivariate models. Regression diagnostics performed to check for collinearity across variables indicated a correlation between two of the covariates used in the model (gestational age and uterine evacuation technology). Nevertheless, in order to ensure that our models accounted for clustered data by site, we estimated models adjusted for individual facilities only, and, in the end, determined to report results from these tests. Our a-priori significance level was set at α = 0.05.

## Results

### Who are PAC clients?

**Fig. 2:**
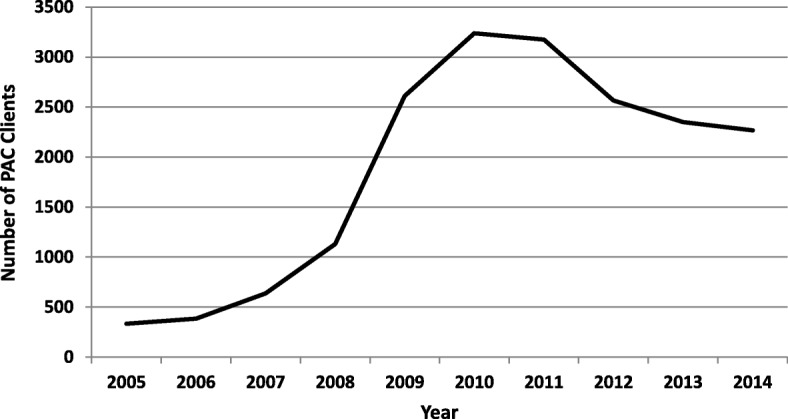
Number of clients utilizing PAC services, by year, Tanzania, 2005–2014

From 2005 to 2014, the 120 facilities admitted a total of 18,688 with incomplete abortions. PAC utilization trends are illustrated in Fig. 2, which shows how scale-up achieved prominent increases in access to services between 2008 and 2010.

Clients’ ages varied widely, from 10 to 54, and the mean age was 27; however, the client distribution across age-groups spread out evenly during the period assessed (Table 1). Roughly, four out of five PAC clients were either married or living in union with their partner at the time of receiving PAC. Parity distribution followed similar contours. Ten percent of clients had no children at the time they received PAC, while 41% of clients had a parity of four or higher. The mean parity for clients under 25 was 1.6; among clients aged 25–29, 3.3; among clients aged 30–34, 4.8; and among clients aged 35 and older, 6.3. Seventy-eight percent of clients received PAC during the first 12 weeks of their pregnancy and nearly 10% after 18 weeks of being pregnant. Over three-fourths of clients received vacuum aspiration, and roughly one-fifth of clients received sharp curettage. Forty-nine percent of clients received PAC at a hospital, 34% at an intermediate-level health center, and 18% at primary-level dispensaries.

**Table 1:** Characteristics of PAC Clients admitted at 120 facilities in Geita and Mwanza Regions, 2005–2014

	No.	%
Age		
<20	2,337	12.5
20–24	5,047	27.0
25–29	4,405	23.6
30–34	3,449	18.5
>35	3,256	17.4
Missing data	194	1.0
Parity		
0	1,877	10.0
1	2,424	13.0
2	3,017	16.1
3	3,026	16.2
4	2,382	12.8
>5	5,253	28.1
Missing data	709	3.8
Gestational age (weeks)		
<12	14,491	77.6
13–18	2,604	13.9
>19	1,125	8.5
Missing data	468	2.5
Uterine evacuation technology		
MVA/EVA^iii^	14,362	76.9
Sharp curettage	3,903	20.9
Missing data	423	2.5
Facility type		
Hospital	9,094	48.7
Health center	6,256	33.5
Dispensary	3,338	17.9
Missing data	0	0

### Trends of decentralization

**Fig. 3:**
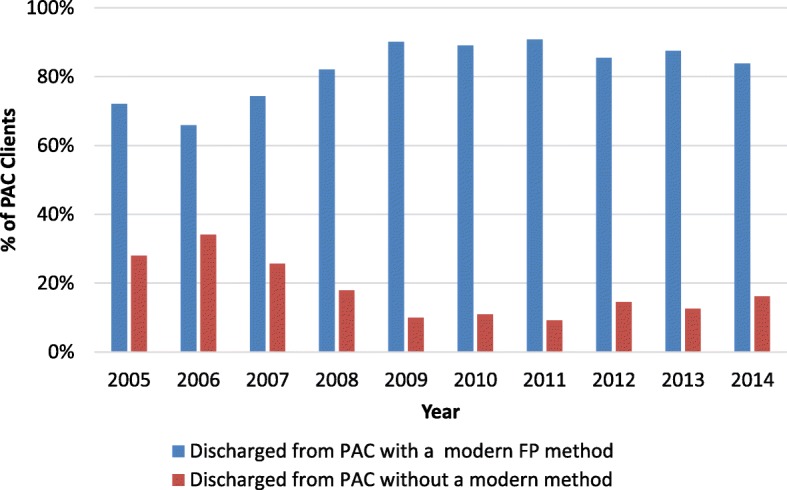
Percentage of PAC clients taking up a modern FP method or adopting no modern method, 2005–2014.

**Fig. 4:**
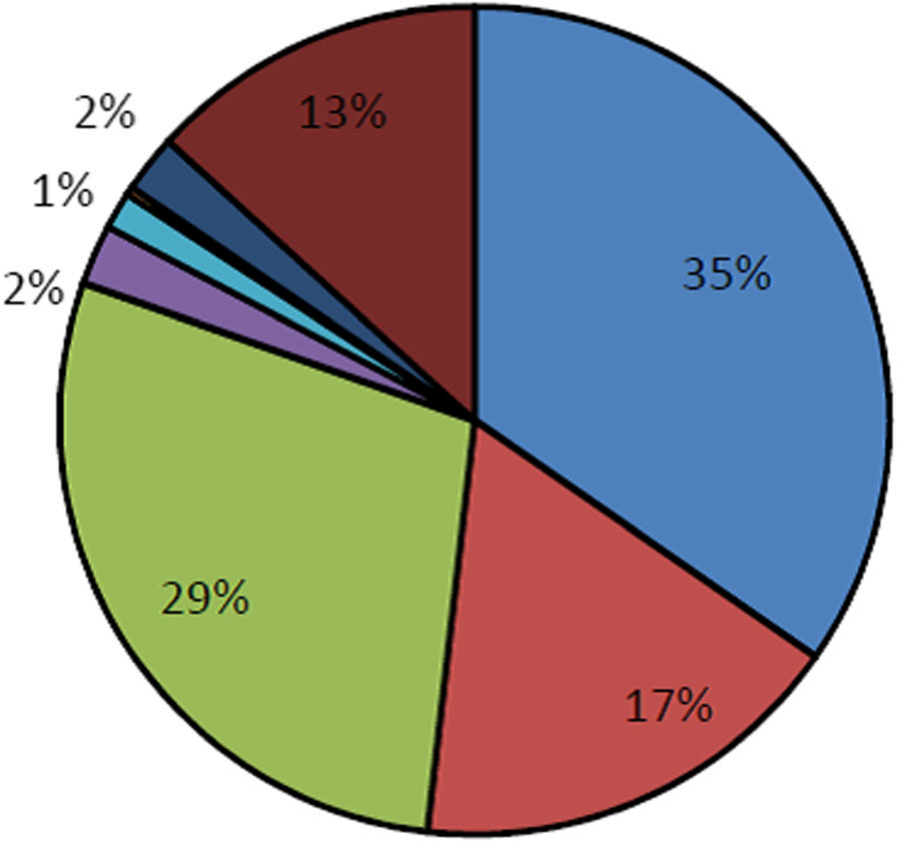
Percentage distribution of PAC clients, by contraceptive method adopted, 2005 to 2014

The proportion of those admitted and adopting a modern FP method (i.e. condom, oral contraception, injectable contraception, implant, IUD and permanent methods) increased from 72% in 2005 to 88% in 2013 (*p* = .04), then slightly decreased to 83% in 2014 (Fig. 3). Over the entire period 2005–2014, 86.5% of all clients received a modern contraceptive method—80.4% a short-acting method (oral contraceptives [35%], injectables [29%], and condoms [17%]) and 6.1% a LARC/PM (Fig. 4). Thus, the vast majority of method adopters selected a short-acting method, a trend that was consistent throughout the scale-up. See Figs. 3 and 4.

**Fig. 5:**
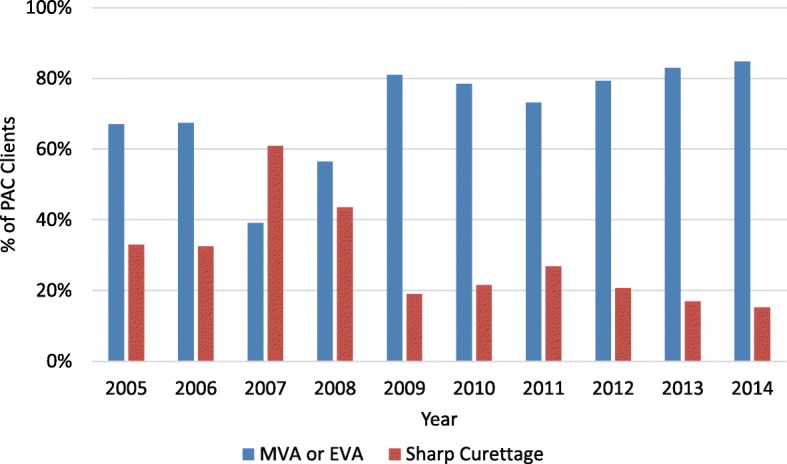
Percentage of PAC clients provided MVA versus sharp curettage, 2005–2014

By 2014, MVA came to supplant sharp curettage as the predominant treatment methodology for incomplete abortion. Whereas in sharp curettage was used to treat roughly 60% of PAC cases in 2007, by 2014 it was used during 17% of cases (*p* = .014). See Fig. 5.

**Fig. 6:**
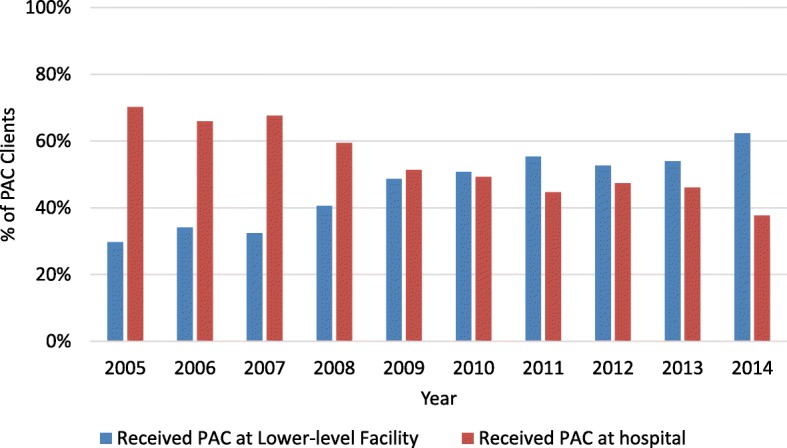
Proportion of PAC clients admitted at a lower-level facility (health center and dispensary)

Lower-level health facilities (health centers and dispensaries) came to provide the majority of PAC services over the course of decentralization, which helped address earlier trends which reflected the disproportionally high utilization of high levels of utilization at hospitals. Encouragingly, this coincided with incremental increases in access to PAC overall (Fig. 6). Between 2005 and 2014, the proportion of clients accessing PAC at these facilities doubled, from 30 to 60% percent (*p* = .004). Nevertheless, it is worth noting that, although primary care dispensaries comprised two-thirds of all facilities to which PAC was expanded, between 2005 and 2014, these facilities never received more than 15% of all PAC cases per year during this period. Furthermore, differences in the proportion of all PAC cases received dispensaries between years during this period were never statistically significant.

### Factors associated with postabortion contraceptive uptake by PAC clients

Table 2 shows results from models developed to test for associations between facility level, client age, gestational age, uterine evacuation method, and contraceptive uptake, estimating the odds of clients’ adopting any modern contraceptive method prior to discharge from PAC services. In general, contraceptive uptake differed by age-group; however, the adjusted odds of any method uptake by PAC clients differed significantly by age, with clients 35 years of age and over (*n* = 3256) less likely than those younger than 20 to adopt a method (OR = 0.82, CI 0.71–0.95). Clients’ uptake of a modern contraceptive method also significantly differed across facility levels, with the adjusted odds of uptake significantly greater in intermediate (OR = 1.41, CI 1.28–1.56) and primary care facilities (OR = 1.19, 1.06–1.34) than at tertiary facilities. Furthermore, the odds of FP adoption were significantly higher among clients whose abortion complications were treated with vacuum aspiration relative to those receiving sharp curettage (OR = 1.98, CI 1.81–2.17). Clients admitted for PAC had significantly lower odds of adopting a modern FP method if their pregnancy was terminated between 13 and 18 weeks of gestation (OR = 0.76, CI 0.67–0.85) and after 19 weeks (OR = 0.57, CI 0.50–0.65).

**Table 2:** Bivariate associations for any PAC FP uptake, and multivariate odds of any PAC FP uptake

	Bivariate associations (chi^2^)		Odds ratio (multinomial logit regression)		
	No.	%	*p*-value	OR	95% CI
Age	18,494		.002		
<20	2,337	12.5		Reference	
20–24	5,047	27.0		1.10	0.95–1.26
25–29	4,405	23.6		1.15	1.00–1.33
30–34	3,449	18.5		1.06	0.92–1.24
>35	3,256	17.4		0.82*	0.71–0.95
Facility type	18,688		.004		
Hospital	9,904	48.7		Reference	
Health center	6,256	33.5		1.41**	1.28–1.56
Dispensary	3,338	17.9		1.19*	1.06–1.34
Uterine evacuation technology	18,465		.01		
MVA/EVA	14,362	76.9		1.98**	1.81–2.17
Sharp curettage	3,903	20.9		Reference	
Gestational age (weeks)	18,220		.003		
<12	14,491	77.6	.003	Reference	
13–18	2,604	13.9		0..76**	0.67–0.85
>19	1,125	8.5		0.57**	0.50–0.65

*p<.05; **p=<.001

Table 3 shows the same analysis, but using PAC clients’ adoption of a LARC or PM as the dependent variable. Relative to adolescents, clients from all ages had significantly higher odds of LARC/PM uptake, which increased in magnitude according to age. Unlike any method adoption, the odds of using a LARC or PM were significantly lower among clients treated for an incomplete abortion at an intermediate health center (OR = 0.69, CI 0.60–0.79). As seen in the analysis in Table 2, clients who received vacuum aspiration had higher odds of adopting a LARC or PM (OR = 1.38, CI 1.17–1.63), while adoption of a LARC or PM was significantly reduced among those admitted at 13–18 weeks into their pregnancies (OR = 0.83, 0.69–0.1.00) or 19 weeks or later (OR = 0.81, CI 0.64–0.99).

**Table 3:** Bivariate associations for PAC LARC/PM uptake, and multivariate odds of PAC LARC/PM uptake

	Bivariate associations (chi^2^)		Odds ratio (multinomial logit regression)		
	N	%	*p*-value	OR	95% CI
Age	18,494		0.04		
<20	2,337	12.5		Reference	
20–24	5,047	27.0		1.56*	1.16–2.10
25–29	4,405	23.6		2.23**	1.67–2.98
30–34	3,449	18.5		3.11**	2.33–4.16
>35	3,256	17.4		6.17**	4.68–8.16
Facility type	18,688		0.003		
Hospital	9,904	48.7		Reference	
Health center	6,256	33.5		0.69**	0.60–0.79
Dispensary	3,338	17.9		0.95	0.81–1.12
Uterine evacuation technology	18,465		0.004		
MVA/EVA	14,362	76.9		1.38**	1.17–1.63
Sharp curettage	3,903	20.9		Reference	
Gestational age (weeks)	18,220		0.002		
<12	14,491	77.6		Reference	
13–18	2,604	13.9		0.83*	0.69–0.1.00
>19	1,125	8.5		0.81*	0.64–0.99

*p<.05; **p=<.001

## Discussion

Overall, PAC clients appear to represent a broad spectrum of women of reproductive age in the general population in terms of key social and demographic characteristics. The decentralization of PAC from tertiary hospital facilities to lower-level facilities proved to have effectively addressed the disproportionally high levels of utilization at the former by increasing the availability of the service in the latter. Postabortion contraceptive uptake (86%) in the 120 facilities evaluated was maintained without significant variation during the period of scale-up. Variables associated with significant differences in postabortion contraceptive uptake were facility level, age, gestational age at the time of treatment, and uterine evacuation technology used.

### Who are PAC clients?

This analysis complements existing sources of data and discussion on the demographic characteristics of women that have had abortion complications and sought PAC. A global meta-analysis of postabortion FP behaviors in developing-country settings found that 47% of clients were above the age of 25 [21]; this study finds the proportion of such clients in Tanzania to be somewhat greater (60%). The finding is consistent with those from more in-depth analyses of women’s decision to continue with unintended pregnancies in high-income settings, which demonstrate that factors associated with relatively later phases in women’s life cycle (including number of living children, marital status and cohabitation with partners, and years since last delivery) are determinants of induced abortion [22, 23]. A detailed analysis of demographic surveillance data in Bangladesh supports the finding that higher maternal age, shorter birth intervals, and advanced parity are determinants of induced abortion; however, very young maternal age is also associated with a greater likelihood of pregnancy termination [24]. The univariate breakdown of the demographic characteristics of 18,688 PAC clients suggests, albeit illustratively, that in Tanzania, demand for PAC is prominent among women at all phases of the reproductive life course, mostly among those at advanced stages of parity. Not surprisingly, the vast majority of clients sought PAC within the first trimester of their pregnancy and were treated with MVA.

### Trends of decentralization

Trends in the utilization of PAC services at the 120 facilities indicate that, by 2010,the scale-up resulted in an increase in utilization of PAC, mostly at lower-level facilities. This reflects a promising departure from earlier patterns of disproportionally high levels of service utilization at regional and district hospitals. A deeper look at decentralization trends, illustrated in our findings section, however, illuminates challenges in shifting treatment seeking for PAC to the primary care level, which is typically closest to communities where women first identify their need for care. This finding may be contextually specific to the local health system in the Lake Zone of Tanzania during this period.

Nevertheless, the challenge we observed promoting treatment seeking for PAC at dispensaries is consistent with results of other efforts to scale up “high-impact practices” at the primary care level. Altogether this points out the need for continuous strengthening of overall health systems that promotes local government coordination, and improves supply chains, training and supervision systems, particularly at the primary care level, manpower and logistics problems are often the most acute [25, 26]. It is encouraging, however, that the delivery of postabortion contraceptive services remained an integral component of the PAC model during scale-up. Similar experiences have been recorded in other settings, such as in Guatemala [27] and in sites supported by Ipas in Sub-Saharan Africa and South Asia, which reported high levels of FP uptake over similar time frames [21]. Though our analysis adds to an emergent body of literature on uptake of modern contraception immediately after PAC, the evidence on long-term postabortion contraceptive continuation remains thin and inconclusive [28, 29].

### Factors associated with postabortion contraceptive uptake by PAC clients

This analysis suggests that the efforts described in this paper contributed to high contraceptive uptake in an environment of low contraceptive prevalence [30, 31]. While encouraging, this study illuminates priorities for future interventions to improve uptake through increased method choice and access.

Overall, uptake of LARCs and PMs is relatively low in the observed sites, particularly among younger women. In Tanzania, the contraceptive method mix is limited by commodity availability in the public sector. For example, implants and IUDs are not always available in lower level facilities, especially frontline dispensaries. These service delivery points also face critical shortages in staff competent at inserting and removing these methods. This can influence providers to prefer delivering short-acting methods [32]. Conversely, other studies report that women prefer short-acting contraception regardless of the availability of a wide method mix in settings where they seek care [33]. Nonetheless, it stands to reason that if the full range of methods were available at all sites, along with providers trained to deliver them, this could increase uptake among PAC clients, generate demand among new users, and allow PAC clients to choose the contraceptive method best aligned with their fertility intentions [34].

Our models estimated the unadjusted and adjusted odds of adopting any modern method or an LA/PM among all of the clients recorded in PAC registers in the 120 sites observed. After conducting regression diagnostics, we elected to report on tests adjusted for clustering by site (Tables 2 and 3). We learned that postabortion uptake of any modern method increased with age with the exception of clients age 35 and older, who were significantly less likely to adopt a modern method relative to clients under 20. Conversely, the adjusted odds of PAC clients’ adoption of an LA/PM increased incrementally according to age, with PAC clients aged 35 and older the most likely to adopt these contraceptive methods. While the associations between age progression and adoption of LA/PMs are consistent with existing knowledge, the negative odds of adopting any modern method among women of advanced age, relative to the youngest women, is at variance with findings from recent studies [35, 36]. This association merits further investigation specific to the service provision, fertility, and method preferences of PAC clients in Tanzania.

As has been observed in other studies, the odds of postabortion uptake of any modern method in our analysis were significantly greater in primary and intermediate facilities than in hospitals [37]. This trend was reversed when the analysis was narrowed to uptake of LARCs/PMs, which PAC clients were significantly more likely to adopt at tertiary facilities. Sterilization services, IUD, and implant services require equipment, commodities, and clinical skills that are, frequently, not available at lower level facilities. Nevertheless, relative to hospitals, health centers and dispensaries receive fewer clients, availing providers more time to counsel clients on fertility awareness and contraceptive methods. Furthermore, they are smaller, and rarely require complex internal referrals. Future improvements should address these imbalances. For example, by reorganizing services at higher-level facilities and by training and motivating providers there to deliver quality, client-centered postabortion counseling, it is likely that programs would facilitate improvements in provision of FP methods during treatment of abortion complications. Similarly, training mid-level providers to deliver IUD, implant, and sterilization services, in addition to uterine evacuation, would improve the availability and quality of services at primary care facilities, leading to more even levels of PAC utilization at higher- and lower-level facilities, respectively [38].

The adjusted odds of adopting any modern contraception were significantly greater among PAC clients who received MVA. Similarly, the likelihood of modern method uptake was significantly less among PAC clients admitted for treatment beyond 12 weeks of gestation. The same associations were observed when the analysis was narrowed to looking at PAC clients’ uptake of LA/PMs. It bears noting that 94% of all sharp curettage cases and 87% of all PAC cases admitted after 12 weeks of pregnancy occurred at hospitals. This suggests that the challenges in delivering postabortion FP services at advanced gestational stages, or when MVA is not used, may stem from problems with the service delivery environment in hospitals, as discussed above and in recent literature [39].

### Limitations

The analysis presented was undertaken post-hoc to capture the results of the PAC decentralization program in Tanzania. Poor collection of PAC service statistics before the intervention precluded us from comparing pre- and post-intervention results. Furthermore, it is possible that data collection was poorly conducted at the enrolled sites by the providers who recorded service statistics.

PAC logbooks did not capture critical information related to clients’ decision to adopt a contraceptive method. This included their fertility intentions, their prior experience of an abortion, the time since their last pregnancy and delivery, the quality of care, and other contextual factors known to influence women’s contraceptive calculus at all stages in their reproductive lives. Furthermore, they do not include information on spousal involvement in FP counseling or whether a spouse/partner adopted FP after PAC. We also only included women served at the public-sector facilities where EngenderHealth provides technical assistance. This does not reflect the situation or trends of women and none served at private-sector sites.

## Conclusion

The experience of expanding PAC services in Tanzania suggests that integrating contraceptive care with treatment for abortion complications can increase FP use. Furthermore, it illustrates how the integrated service delivery model can remain effective during scale-up. These findings are in keeping with the evidence that has led to global recognition of PAC as a high-impact practice [14]. Yet there are numerous examples of international and country-level policies that undermine efforts to help women realize their reproductive intentions and protect their health, particularly after an unsafe abortion [35, 40]. Where policies are enabling, this may be undermined by weak health systems and implementation capacity to address the particular needs of PAC clients who have expressed a demand for FP. Thus, there are opportunities to apply the experience reported in these pages to the effect of increasing contraceptive access and choice to women in this situation. If implemented methodically and at scale, this may strengthen systems and make gains against preventable maternal mortality [41].

Method choice and uptake in postabortion settings is influenced by a range of factors related to clients’ socio-demographic characteristics, their individual preferences, their familial and social context, provider knowledge and attitudes, the strength of local health systems, and population policies. Only some of these could be examined in this analysis; much remains to be understood from research that can draw upon richer data sources than routine service statistics. Nevertheless, it is clear that by collaborating with governments, organizations can foster critical change. If partners work together, with a vision of reorganizing services, increasing access to new methods at all levels of care, training providers, and strengthening management systems, they can effectively reposition FP and address unmet need and its adverse sequelae on postabortion women, both in Tanzania and in other settings where the need for such interventions is acute.

## Abbreviations

CI: Confidence Interval
EVA: Electric vacuum aspiration
FP: Family planning
IUD: Intra-uterine device
LA/PM: Long acting permanent method (of contraception
LARC: Long acting reversible contraception
MoHGCDEC: Ministry of Health, Gender, Community Development, Elderly and Children
MVA: Manual vacuum aspiration
OR: Odds ratio
PAC: Postabortion care
PM: Permanent method (of contraception)
UNFPA: United Nations Population Fund
USAID: United States Agency for International Development
WHO: World Health Organization

## Footnotes

1 In 2005, Geita was a district that formed part of Mwanza Region. In 2012, Geita became a separate region.

2 COPE®, which stands for client-oriented, provider-efficient, is a process for improving the quality of health care that was developed by and is used by EngenderHealth in its programs.

3 Eighty-two clients were treated with electric vacuum aspiration (EVA).
